# Effects of simulated litter inputs on plant-microbe carbon pool trade-offs in degraded alpine meadows

**DOI:** 10.3389/fpls.2025.1549867

**Published:** 2025-04-03

**Authors:** Weishan Lin, Kejia De, Xuemei Xiang, Tingxu Feng, Fei Li, Xijie Wei

**Affiliations:** Academy of Animal Husbandry and Veterinary Medicine, Qinghai University, Xining, China

**Keywords:** Qinghai-Tibet Plateau, alpine meadow, microbial biomass carbon, carbon pool, trade-offs

## Abstract

**Introduction:**

Litter, as a major carbon source in alpine meadow ecosystems, seriously affect the variation of plant-microbe carbon pools in alpine meadows. In order to study the response of plant-microbial biomass carbon pool trade-offs in degraded alpine meadow to litter inputs.

**Methods:**

We investigated the effects of different levels of litter inputs on the carbon pools of alpine meadows plant aboveground communities, the carbon pools of the root, and the total carbon pools of the plant communities and the soil microbial biomass carbon pools, and clarified the variable factors that affect the balance of the plant-microbial biomass carbon pools and the process of influencing the trade-offs.

**Result:**

(1) Litter inputs had a positive effect on plant carbon pools, and the aboveground community carbon pools, root carbon pools, total plant community carbon pools and soil microbial biomass carbon pools of alpine meadows were all maximized in the T3 treatment. (2) The trade-off analyses showed that the trade-off relationships of ungrazed alpine meadows TPCPMBCP in the following order under different levels of litter treatments: T1(0.0414) > T2 (0.0269) > T0 (0.0086) > T3 (0.0012), the trade-off relationship of TPCP-MBCP in lightly grazed alpine meadows was in the order of T2 (0.0494) > T3 (0.0140) > T0 (0.0097) > T1 (0.002), and the tradeoff relationship of TPCP-MBCP in moderately grazed alpine meadows was in the order of T3 (0.0383) > T1 (0.0307) > T2 (0.0196) > T0 (0.0005). (3) Propensity analysis showed that the TPCP-MBCP trade-offs tended to favor MBCP under ungrazed, lightly grazed and moderately grazed meadows under the T1 treatment. (4) Structural equation modeling showed that RB and APC were positively correlation, RCP was significantly negatively correlated with the TPCPMBCP trade-off in lightly grazed grassland (*P*<0.05), and MBC was significantly positively correlated with the TPCP-MBCP trade-off in moderately grazed grassland (*P*<0.05).

**Discussion:**

There was no uniform pattern in TPCP-MBCP trade-off and propensities in ungrazed, lightly grazed, and moderately grazed alpine meadows under different levels of litter inputs. This study can help to optimize the grazing management measures, predict the changes of carbon pools in alpine meadows and clarify the transfer and storage of carbon pools between plants and microorganisms, so as to provide a theoretical basis for the study of carbon pools in degraded alpine meadows.

## 1 Introduction

Alpine meadows in China are mainly distributed on the Qinghai-Tibetan Plateau and in the alpine zone of various high mountain systems, with a total area of about 87 million hm^2^, accounting for 22.1% of the national grassland area, which is one of the largest grassland types in China. The sustainable utilization of alpine meadow grassland is directly related to the production and life of herders on the Qinghai-Tibetan Plateau (Wang et al., 2018). As an important part of the global terrestrial ecosystem, alpine meadows are an important carbon source/sink, with soil organic carbon (SOC) reserves of about 33.5 Pg C, accounting for 2.5% of the global SOC pool, but covering only 0.3% of the earth’s land area, and playing a crucial role in the global carbon cycle (Breidenbach et al., 2022; Friedlingstein et al., 2020; Yang et al., 2015). Carbon stocks in the alpine grasslands of the Tibetan Plateau may have significant long-term impacts on the global carbon cycle (Yang et al., 2011). However, the Tibetan Plateau is a sensitive and critical area for global climate change, which affects biogenic carbon by controlling the composition of plant communities, the distribution of above-ground and below-ground inputs to the soil, the composition of microbial communities and biogeochemical processes. The Sanjiangyuan, known as the “Water Tower of China”, is the birthplace of the Yellow, Yangtze and Lancang Rivers, and plays an important role in water conservation and maintaining species diversity. The alpine grasslands of the Qinghai-Tibet Plateau are the main grazing grasslands for native herbivores, and grazing is an important driving force for grassland succession. Grazing is an important driver of grassland succession. Grazing mainly causes plant community composition, structure, survival strategy, and nutrient cycling characteristics through livestock feeding and trampling (Qiao et al., 2009; Wang et al., 2009). In the late 1990s, scholars at both domestic and international level began to pay attention to the degradation of alpine grasslands on the Tibetan Plateau caused by grazing (Lu et al., 2017; Shang et al., 2014; Zhu et al., 2023), The grazing phenomenon has caused a reduction in plant height, cover and biomass, and a decrease in the carbon pool of plant aboveground communities; at the same time, the reduction in surface litter has reduced the amount of research carbon entering the soil, and the carbon pool of soil microbial biomass has decreased as a result.

As a link between above-ground vegetation and below-ground soil, litter plays an important role in nutrient metabolism and cycling in the plant-soil system through different rates of nutrient return and decomposition (Deng et al., 2016). Plant community carbon stocks, also known as carbon pools, are obtained from the product of plant biomass and plant carbon content, and represent the ability of plants to store, absorb, and utilize carbon in the soil (Mao et al., 2021). Plant aboveground community biomass (AGB) determines the carbon pool of aboveground plant communities (APCP). The results of studies of carbon pools in plant aboveground communities vary due to different estimates of aboveground biomass (Butterfield and Malmström, 2009; Flombaum and Sala, 2007; Paruelo et al., 1997; Prince and Tucker, 1986). Below-ground plant carbon pools are the sum of carbon contained in plant root biomass (RB) below the surface of grassland vegetation. Plant root carbon pools (RCP) are an important component of carbon stocks in grassland vegetation. There are many methods for the study of below-ground biomass, such as soil borings, in-growth cores, micro-root zones, nuclear magnetic resonance (NMR), X-ray methods, etc (Ingram and Leers, 2001; Ni, 2001; Zhu et al., 2008), and the different methods have their own advantages and disadvantages. It is estimated that the carbon in the alpine grasslands of the Qinghai-Tibetan Plateau (including vegetation and soil) accounts for about 54.5% of the total carbon in grasslands in China (Ni, 2002). However, due to spatial and temporal variability and differences in the methods used by researchers, the carbon pools of alpine meadows plants have not yet been standardized.

Soil microbial biomass carbon (MBC) is the most active and changeable part of soil organic matter, although it only accounts for 0.3%~9.9% of the total soil carbon, but it is the driving force of soil organic carbon and nutrient transformation and cycling, directly involved in the decomposition and transformation of organic carbon, and is an important source of soil nutrient reserves and nutrients for plant growth (Wang et al., 1996; Wen et al., 2004). Litter input and removal treatments have been widely used as an effective experimental method for evaluating the effects of litter on soil microbial biomass and community structure in terrestrial ecosystems (Liu et al., 2019; Wang et al., 2017). The conventional wisdom is that increased inputs of litter will increase soil microbial biomass carbon (Pan et al., 2018; Pioli et al., 2020), causing a change in microbial structure (Jin et al., 2010). Leff et al., 2012 observed a significant reduction in soil MBC content in apoplastic removal treatments, which was confirmed by Meta-analysis results of Xu et al., 2013. Soil microbial biomass carbon pools (MBCP) are the sum of carbon contained in the bodies of all microorganisms in the soil, including bacteria, fungi, actinomycetes, protozoa and algae. Therefore, changes in MBC affect the content of MBCP. However, fewer studies have been reported on the effects of litter on plant carbon pools and MBCP in alpine meadows, and in particular, studies related to litter trade-offs between plants and microorganisms in degraded alpine meadows on the Tibetan Plateau have not been reported.

Therefore, in this study, we chose alpine meadows as the research object, and set up experiments with different levels of litter input (F0 (CK)), 20% of the standardized amount (F1), 50% of the standardized amount (F2) and 100% of the standardized amount (F3) by simulating the effects of different levels of litter inputs on plant-microbial carbon pools of alpine meadows with ungrazed meadows (sealing), lightly and moderately grazed alpine meadows, to analyze plant-microbial carbon pool trade-offs and propensity analysis of carbon pools under different levels of litter inputs. We focus on the following scientific issues: (1) What are the dynamic changes of APCP, MBC and MBCP under different litter treatments? (2) to analyze the plant-microbial carbon pool trade-off relationship under different litter treatments, and clarify whether the carbon pool under litter treatments contributes to the total plant community carbon pool (TPCP) or the MBCP? and (3) to explain the key factors affecting the carbon pool trade-offs of alpine meadow under litter inputs through the random forest model, and constructing structural equation models. This study will help researchers working on the mechanism of litter on plants and microorganisms to crystallize their scientific problems, and will contribute to the scientific management of the Qinghai-Tibetan Plateau and the rational use of grassland resources.

## 2 Materials and methods

### 2.1 Study area

This study was carried out in accordance with the construction of the sub-station of the National Field Scientific Observation and Research Station of Grassland Ecosystem of Sanjiangyuan, Qinghai, China. Referring to the classification standard of Technical Regulations for Yak Grazing Utilization in Alpine Meadows (DB63/T607-2006) issued by Qinghai Bureau of Quality and Technical Supervision (QTS) (Wang, 2005), which was drafted by Northwest Plateau Biological Research Institute of Chinese Academy of Sciences (NWPBRI) and Qinghai Academy of Animal Husbandry and Veterinary Medicine (QAAVM), based on the criteria of dominant species of the plant community, graminoid cover, and so on (**Table 1**), the grassland was classified as ungrazed grassland, lightly grazed grassland, and moderately grazed grassland.

**Table 1:** Classification of alpine grassland degradation and sample sites.

	Longitude and latitude	Altitude (m)	Plant community dominant species	Cover of grasses (%)
No-grazed	97°18′17″E, 33°24′40″N	4238	Gramineae+ Salicaceae+ Weeds	>30
Lightly grazed	97°18′17″E, 33°24′36″N	4270	Gramineae+ Salicaceae+ Weeds	20-30
Moderated grazed	97°20′50″E, 33°24′15″N	4255.8	Gramineae+ Salicaceae+ Weeds	20~30

### 2.2 Experimental design

In order to investigate the effects of different levels of litter inputs on the plant-microbe carbon pool trade-off in alpine meadows under different grassland utilization methods, the present study was carried out by selecting sample plots in the study area. Due to the perennial low temperature and long decomposition process of plant litter on the Tibetan Plateau, this study chose to use glucose instead of plant litter for the simulation experiment (Lin et al., 2025). The area of each plot was 1 m^2^ (1 m×1 m). In grassland ecosystems, more than 90% of the net production of plants is returned in the form of litter (Fanin and Bertrand, 2016; Loranger et al., 2002). In consideration of this, this study is based on the previous research results of alpine meadows in the Sanjiangyuan area (Rumpel et al., 2018), as well as combining the current situation of the experimental area. Meanwhile, referring to the published literature (Xiao et al., 2021; Zhou et al., 2021). The glucose addition in this experiment was based on 2% APC (Aboveground Community Carbon Content of Vegetation). Four treatments were set up in this study, 20% of the standard amount (T1), 50% of the standard amount (T2), 100% of the standard amount (T3) and T0 (CK). In the experiment, each experimental plot was separated by a 20-mm-thick polyethylene material in order to prevent the flow of the saccharose solution between the different plots (the depth of the partitions was about 60 cm). The glucose from each treatment was dissolved in 3L of tap water and shaken well to ensure that no solute remained in the beaker and spray bottle. After shaking well, the glucose solution was evenly sprayed on the surface of the test sample with a small spray bottle (Gao, 2022). The plot area was 1m × 1m = 1m², the plot interval was 1 m, and there were 4 replications, totaling 4×4 = 16 plots. Three types of grassland, such as fenced (F), lightly grazed (L) and moderately grazed (M), totaled 48 plots.

### 2.3 Measurement program and method

Samples were collected at the end of July 2024 during the plant growing season in the above test area.

#### 2.3.1 Above- and below-ground biomass collection of plant communities and their determination

In the experimental area, we chose flat terrain, evenly distributed plants and representative grassland as the experimental sample plots, and set up sample squares of 1 m × 1 m for plant and soil sample collection. Aboveground plants were mowed flush with the ground, packed into envelopes and placed in a cool place; belowground biomass was collected from 0-30 cm soil layer using a soil auger with an inner diameter of 5 cm, and the samples were packed into envelopes and bags, brought back to the laboratory to remove gravel and sand, and then the roots were separated out using a standard soil sieve with an aperture of 0.28 mm and rinsed and air-dried, and the aboveground and belowground biomass were put into the oven to be dried at 60°C for 48 h until constant weight, and then weighed. Above-ground and below-ground biomass were dried in an oven at 60°C for 48h and weighed (Lin, 2023). Samples were taken five times mixed to make one sample. Six replicates were made.

Soil samples were collected by soil auger method (Lin et al., 2024) from 0~30 cm soil layer in the sample plots where the aboveground plant characteristics had been sampled, and the soil samples were mixed five times with soil auger with inner diameter of 5 cm in each sample plot to form a single sample. 5 replicates were made. The soil samples were transported back to the laboratory for mixing and sieving, and then air-dried in a cool and ventilated place for the determination of soil total nitrogen and organic carbon content.

#### 2.3.2 Measurement of plant carbon content

Weighed above-ground samples of plants and below-ground roots were pulverized with a ball mill MM400, sieved through a 200mesh sieve, and community-level carbon content was determined using an elemental analyzer (FLASHAMART) (Lv et al., 2024).

#### 2.3.3 TPCP is calculated using the following formula:


$$\text{TPCP}\left(\text{g}/{\text{m}}^{2}\right)=\text{B}\times \text{C}/1000$$

In the formula, TPCP, B and C represent the total plant carbon pool (g/m^2^), biomass (g/m^2^) and carbon content (mg/g), respectively.

#### 2.3.4 Soil microbial biomass carbon measurement

Within each replicate plot under different grazing treatments, soil samples were taken in layers of 0-10 cm, 10-20 cm and 20-30 cm, using a soil auger with a diameter of 4 cm, and 3 augers were sampled in each layer within each replicate plot, and then 3 replicate samples from the same level were mixed, and the microbial biomass of the soil layer of 0-30 cm was the sum of microbial biomass of each layer. Soil samples were stored at 4°C under refrigeration for the determination of soil microbial biomass.

#### 2.3.5 Soil bulk weight

Measured by the ring knife method (Huang et al., 2024). The formula is as follows:


$$\begin{array}{c} \text{Soil Bulk Weight }\left(\text{g}/{\text{m}}^{3}\right)\\ =\text{Quality of Dried Soil }\left(\text{g}\right)/\text{Volume of Ring Knife }\left({\text{m}}^{3}\right) \end{array}$$

#### 2.3.6 Measurement of MBC content

Referring to Vance et al. (1987), Determination using chloroform fumigation leaching method. Each soil was divided into two portions, one portion was fumigated in a vacuum desiccator for 24 h, and the other portion was not fumigated. Both fumigated and unfumigated soils were leached with 0.5 mol/L K_2_SO_4_ (water-soil ratio: 4:1, 40 mL of 0.5 mol/L K_2_SO_4_ solution, 10 g of fresh soil), and the leachate was filtered and then sucked up 10 mL to 50 mL bottles, and then analyzed by a TOC analyzer (Warip TOC SELECT) to determine the MBC content. The MBC content was finally calculated from the difference between the fumigated and unfumigated values, with a conversion factor of 0.45 for MBC.

#### 2.3.7 MBCP calculation

TMBCP is calculated by the following formulas (Guo and Gifford, 2002; Li et al., 2014):


$$\text{MBCP}(\text{g}/{\text{m}}^{2})=\text{MBC}\left(\text{mg}/\text{kg}\right)\times \text{BD}\left(\text{g}/{\text{m}}^{3}\right)\times \text{H }\left(\text{cm}\right)\times 10$$

where H denotes the thickness of the soil layer.

### 2.4 Data processing

The relative deviation from the mean is determined by the Root Mean Square Error (RMSE), which is a calculation of the deviation between observations and model predictions that is used to assess the extent of the trade-off between the TPCP and the MBCP. The relative benefits of the TPCP and the MBCP are calculated as:


Astd=(A−Amin)/(Amax–Amin)

where Astd is the relative benefit (i.e., standardized value) of TPCP or MBCP, with a range of 0~1. A is the observed value of TPCP or MBCP, and Amax and Amin are the maximum and minimum values of TPCP and MBCP, respectively.

The trade-off index is calculated as follows:


$$RMSE=\sqrt{\frac{1}{n-1}\sum_{i-1}^{n}{\left(A_{std}-Ā\right)}^{2}}$$

Ā indicates the mean of the observations, RMSE indicates the mean deviation from Ā, and the RMSE values of TPCP and MBCP determine the coordinate positions of the points. If the left side of the point is on the upper left side of the 1:1 trade-off line it indicates that the accumulation of TPCP is favored, and if the coordinates of the point are on the lower right side of the 1:1 trade-off line it indicates that the accumulation of MBCP is favored. The distance from the coordinate point to the 1:1 trade-off line represents the trade-off capacity of either TPCP or MBCP, with the greater the distance the greater the trade-off capacity.

All data were tested for normality and chi-square, and one-way ANOVA and Tukey’s multiple comparisons were used to determine differences in plant biomass, carbon content, and plant community carbon pools, MBC, and MBCP between different litter inputs, with significant differences assessed at the *P ≤ 0.05* level. Random forest analysis was used to screen for impact factors with significant effects on TPCP and MBCP trade-offs. Structural equation modeling was constructed with piecewise SEM package based on the variable factors with significant effects, which was used to explore the direct and indirect effects of TPCP and MBCP trade-offs. All statistical analyses were done in R4.4.2 and statistical graphics were done in Origin Pro 2021.

## 3 Results

### 3.1 Effects of different levels of litter inputs on carbon pools of alpine meadow plant communities

**Figure 1:**
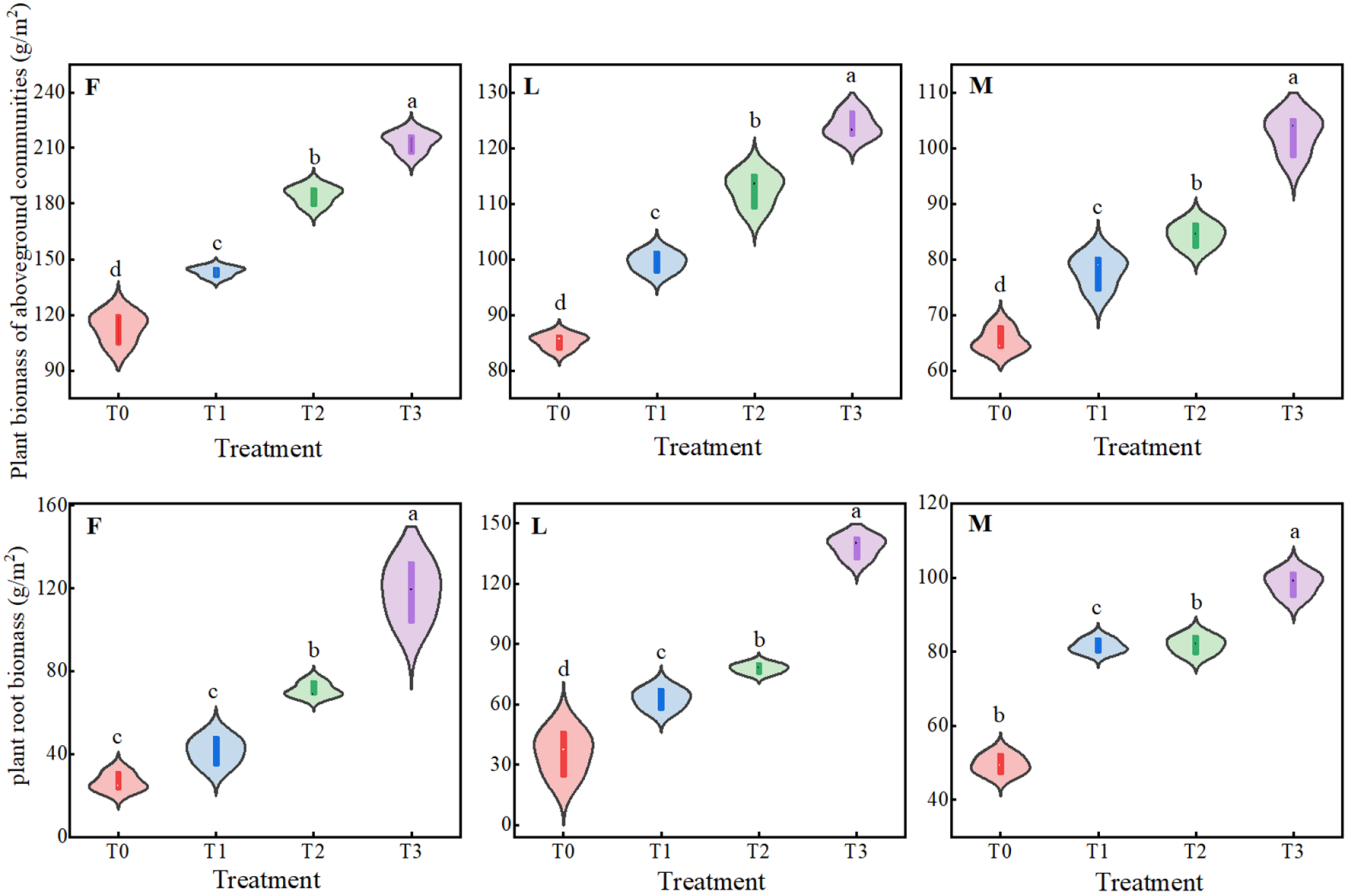
Effects of different levels of litter input on plant biomass in alpine meadows. Different lowercase letters in the figure indicate significant differences between treatments (*P< 0.05*). F = fenced alpine meadows, L = lightly grazed alpine meadows, M = moderately grazed alpine meadows.

As can be seen from **Figure 1**, there were significant dynamic changes in the biomass of closed (*P<0.05*), lightly grazed and moderately grazed alpine meadows under different levels of litter treatments. Among them, the AGB of ungrazed, lightly grazed and moderately grazed alpine meadows showed a similar trend of gradual increase, and all of them had the maximum value at T3, which was 213.35 g/m^2^, 124.01 g/m^2^ and 102.54 g/m^2^, respectively, with a significant difference (*P<0.05*). The trend of RB was similar to that of the dynamic changes of AGB, and all of them had the peak at T3, which was 118.52 g/m^2^, 118.52 g/m^2^ and 102.54 g/m^2^, respectively. were 118.52 g/m^2^, 138.51 g/m^2^ and 98.4 g/m^2^, with significant differences (*P<0.05*).

**Figure 2:**
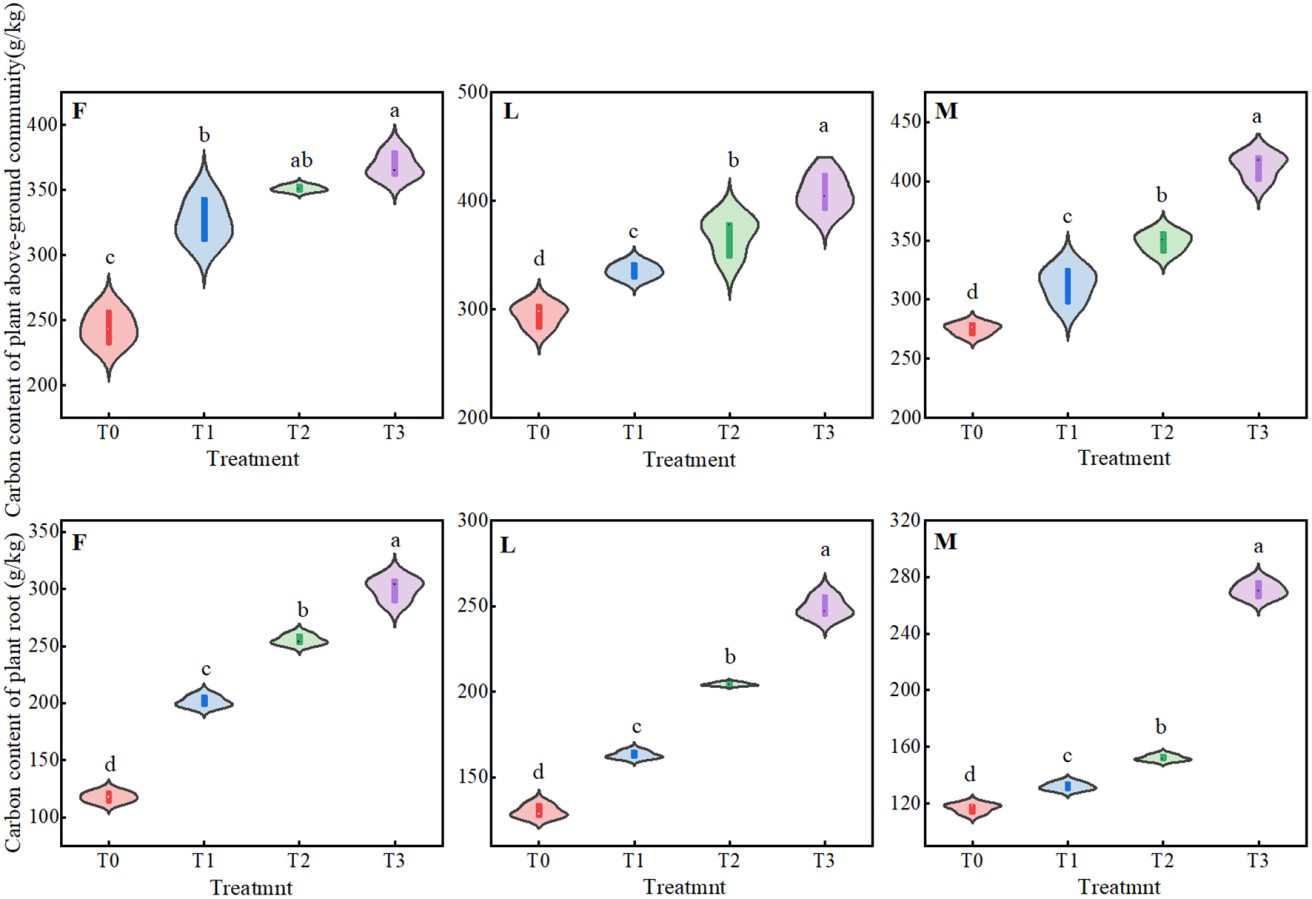
Effects of different levels of litter inputs on plant carbon content in alpine meadows. Different lowercase letters in the figure indicate significant differences between treatments (P < 238 0.05). F = fenced alpine meadows, L = lightly grazed alpine meadows, M = moderately grazed alpine meadows.

As can be seen from **Figure 2**, APC and RC in ungrazed, lightly grazed and moderately grazed alpine meadows had similar patterns of change under different levels of litter treatments, with significant differences (*P<0.05*). APC had its maximum values under T3 treatment, which were 368.43 mg/g, 407.07 mg/g and 412.94 mg/g, respectively; and RC reached its peak value under T3 treatment, which was 300.05 mg/g, 249.23 mg/g and 270.77 mg/g, respectively. 300.05 mg/g, 249.23 mg/g and 270.77 mg/g, respectively.

**Figure 3:**
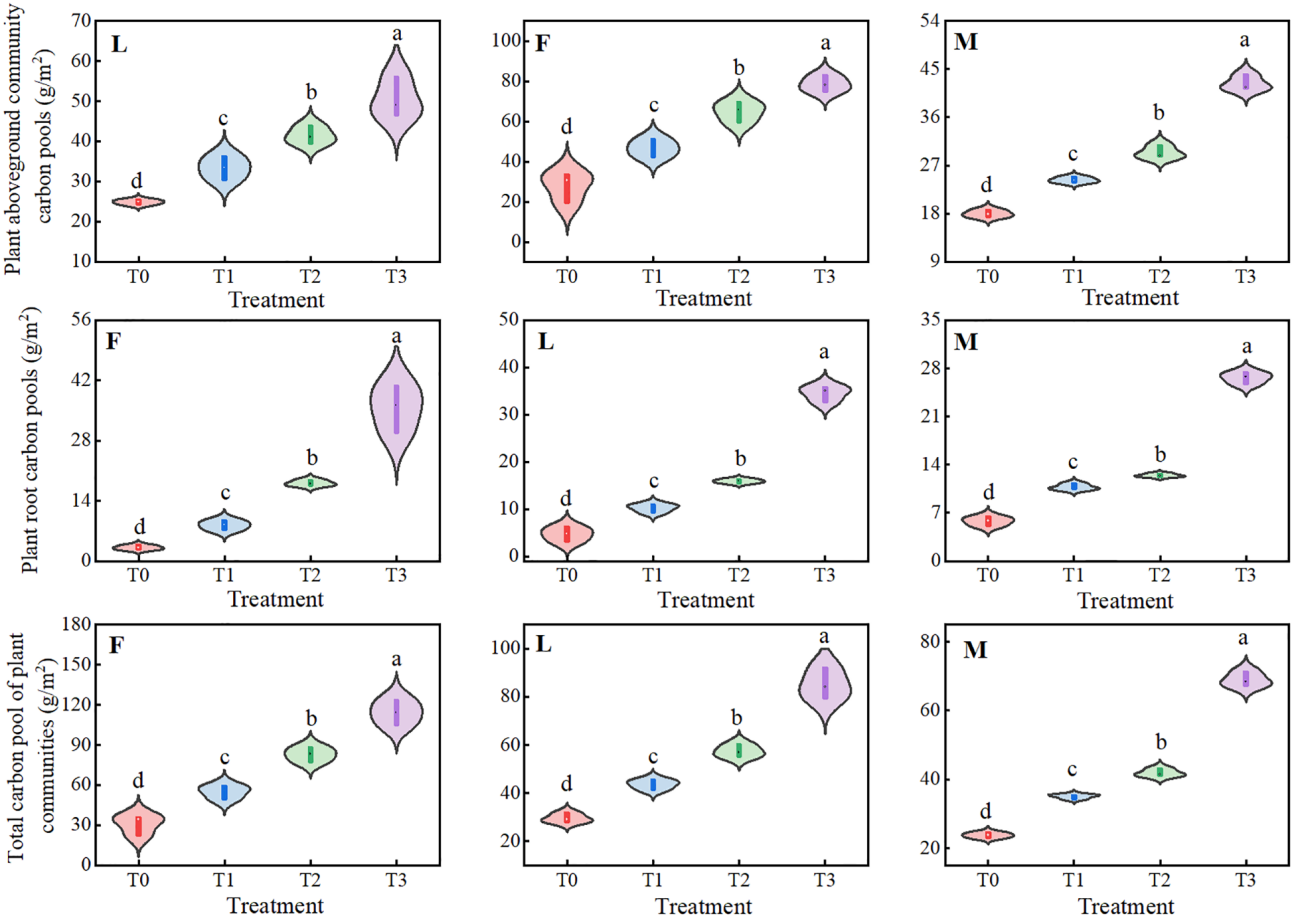
Effects of different levels of litter inputs on plant carbon pools in alpine meadows. Different lowercase letters in the figure indicate significant differences between treatments (P < 238 0.05). F = fenced alpine meadows, L = lightly grazed alpine meadows, M = moderately grazed alpine meadows.

The overall APCP of ungrazed, lightly grazed and moderately grazed alpine meadows under different levels of litter input showed an upward trend of gradual increase, with significant differences (*P<0.05*), and all of them peaked under the T3 treatment, at 78.57 g/m^2^, 50.56 g/m^2^ and 42.33 g/m^2^, respectively. The RCP of ungrazed, lightly grazed and moderately grazed alpine meadows showed an upward trend with the increase of apomictic input, and apomictic treatments significantly increased plant root carbon pools (*P<0.05*), all of which peaked at 35.66 g/m^2^, 34.52 g/m^2^ and 26.23 g/m^2^, respectively, under the T3 treatment. The total plant community carbon pools (TPCP), which is defined as the sum of the aboveground plant community carbon pools and the plant root carbon pools, were not increased in the ungrazed, lightly grazed The trends of TPCP in ungrazed, lightly grazed and moderately grazed alpine meadows were similar to those of APCP, with the overall dynamic changes showing significant increases (*P<0.05*), all of which peaked under the T3 treatment, at 114.23 g/m^2^, 85.08 g/m^2^ and 68.95 g/m^2^, respectively (**Figure 3**).

### 3.2 Effects of different levels of litter inputs on soil microbial biomass carbon pools

**Figure 4:**
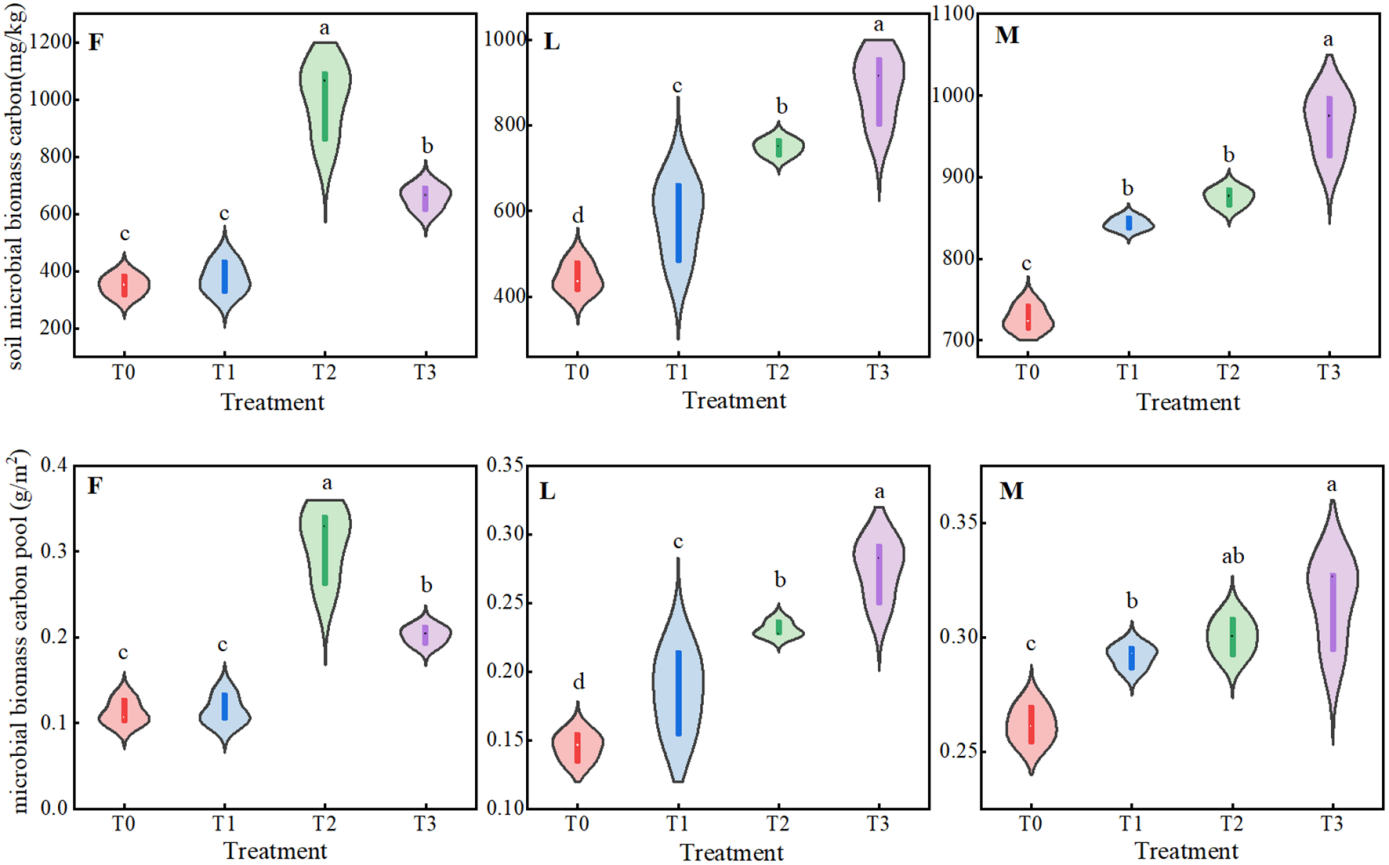
Trade-off between total plant community carbon pool and soil microbial biomass carbon pool under different liter treatments. Different lowercase letters in the figure indicate significant differences between treatments (P < 238 0.05). F = fenced alpine meadows, L = lightly grazed alpine meadows, M = moderately grazed alpine meadows.

As can be seen from **Figure 4**, under different levels of litter treatments, there were significant dynamic changes (*P<0.05*) in MBC of ungrazed, lightly grazed and moderately grazed alpine meadows, and the trend of MBCP was similar to that of microbial biomass carbon content. Among them, MBC and MBCP of ungrazed alpine meadows showed a trend of increasing and then decreasing, both of which had maximum values under F2 treatment, which were 1005.14 mg/kg and 0.20 g/m^2^, respectively. The MBC and MBCP of lightly and moderately grazed alpine meadows increased with the increase of apomictic inputs, and both reached the peak values under F3 treatment, which were 889.68 mg/kg and 0.28 g/m^2^, and 966.02 mg/kg and 0.32 g/m^2^, respectively.

### 3.3 Trade-offs between total plant community carbon pools and microbial biomass carbon pools

**Figure 5:**
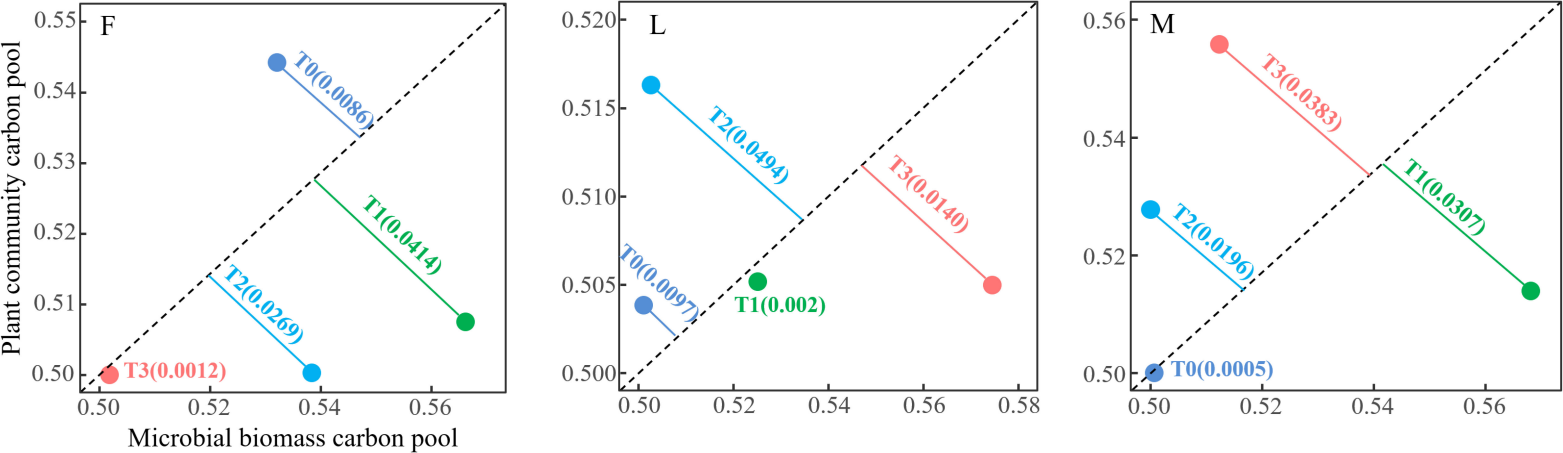
Trade-off between total plant community carbon pool and soil microbial biomass carbon pool under different litter treatments. F = fenced alpine meadows, L = lightly grazed alpine meadows, M = moderately grazed alpine meadows.

From **Figure 5**, it can be seen that there were differences in the trade-off relationship between TPCP and MBCP in three types of alpine meadows: ungrazed, lightly grazed and moderately grazed under different levels of apomictic treatments. The results of trade-off analysis in ungrazed alpine meadows showed that the trade-off relationship between TPCP and MBCP under different levels of litter treatments was in the order of T1 (0.0414) > T2 (0.0269) > T0 (0.0086) > T3 (0.0012), and that the trade-off relationship between TPCP and MBCP under different levels of litter treatments in lightly grazed alpine meadows was in the order of T2 (0.0494)>T3(0.0140)>T0(0.0097)>T1(0.002); the trade-off relationship between TPCP and MBCP under different levels of litter treatments in moderately grazed alpine meadows was in the order T3(0.0383)>T1(0.0307)>T2(0.0196)>T0(0.0005).

The propensity analysis showed that the trade-offs of carbon pools in ungrazed alpine meadows were more in favor of TPCP under the L0 treatment, and MBCP under the L1, L2 and L3 treatments; the trade-offs of carbon pools in lightly grazed alpine meadows were more in favor of TPCP under the L0 and L2 treatments, and MBCP under the L1 and L3 treatments; the trade-offs of carbon pools in moderately grazed alpine meadows were more in favor of TPCP under the M2 and M3 treatments, and MBCP under the M1 treatment; the trade-offs in the M2 and M3 treatments were more in favor of TPCP under the TPCP treatment, and MBCP under the M1 treatment. The trade-offs of carbon pools in moderately grazed alpine meadows were more in favor of TPCP in the M2 and M3 treatments, and more in favor of MBCP in the M1 treatment (**Figure 5**).

**Figure 6:**
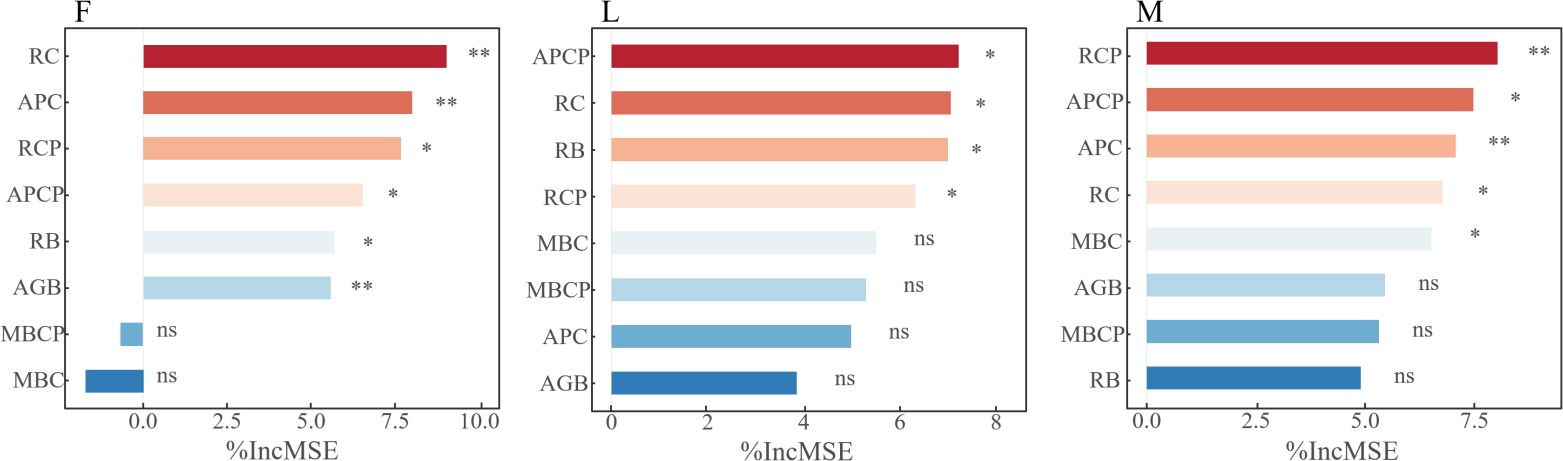
Analysis of factors affecting carbon and nitrogen pool trade-off in plant communities and soil microbial biomass. In the figure, * represents *P< 0.05*, ** represents *P< 0.01*, and ns represents non-significant difference. F = fenced alpine meadows, L = lightly grazed alpine meadows, M = moderately grazed alpine meadows.

The above studies analyzed the dynamic changes of plant biomass, plant carbon content, soil microbial biomass carbon content and carbon pools in different alpine meadows under different levels of apomictic treatments, and in order to further clarify the key factors of plant biomass, plant carbon content and soil microbial biomass carbon content that affect the trade-off between TPCP and MBCP. Through the random forest model analysis, it was found that RC, APC, RCP, APCP, RB and AGB were the main variable factors affecting the trade-off in closed alpine meadows, and all of them reached the significant level (*P<0.05*); APCP, RC, RB and RCP were the main variable factors affecting the trade-off in lightly-grazed alpine meadows, and all of them reached the significant level (*P<0.05*); and the moderately-grazed RCP, APCP, APC, RC and MBC in alpine meadows were the main variable factors affecting trade-offs and all reached significant levels (*P<0.05*) (**Figure 6**).

**Figure 7:**
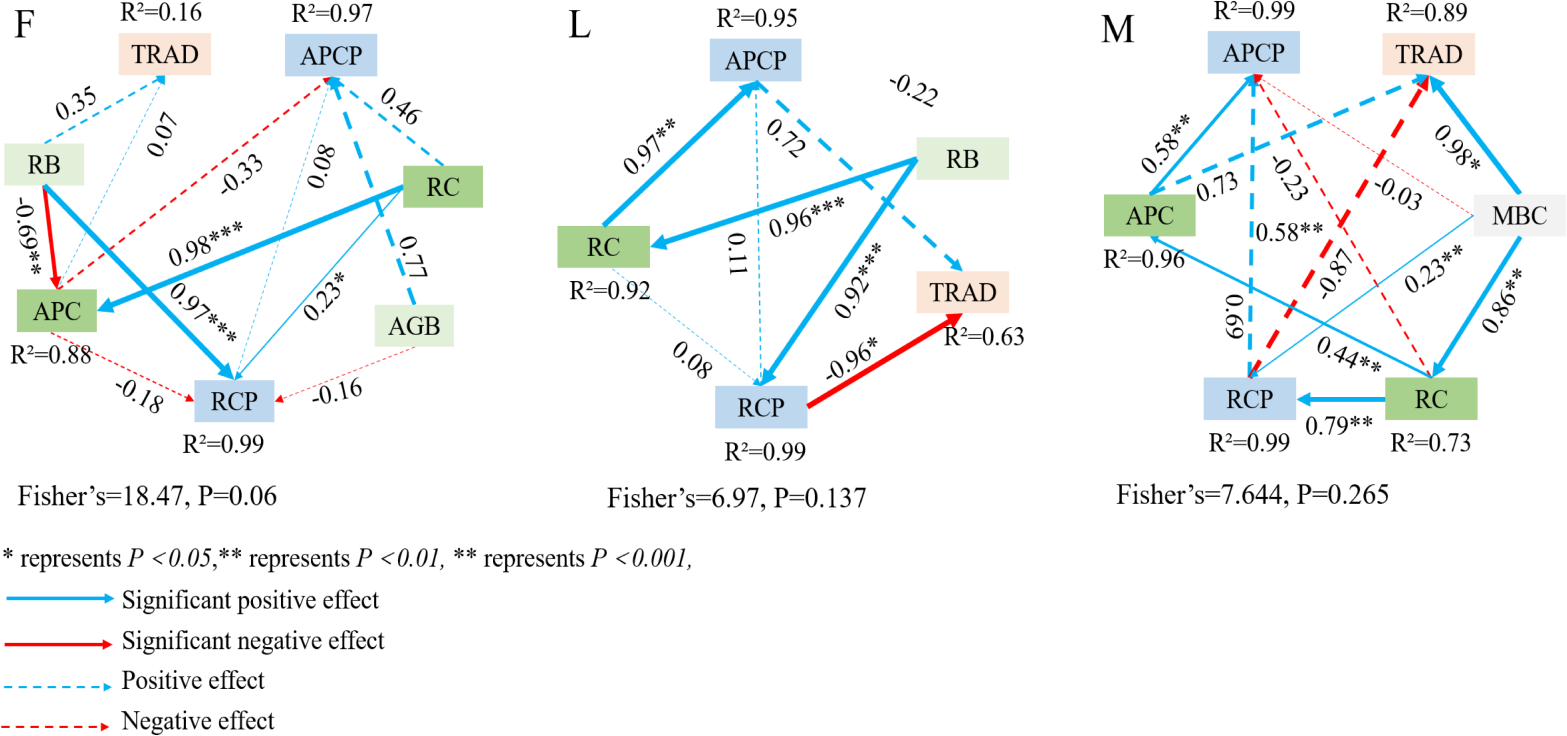
Structural equation modeling analysis of plant and soil microbial biomass carbon pool trade-offs. F = fenced alpine meadows, L = lightly grazed alpine meadows, M = moderately grazed alpine meadows.

The processes affecting TPCP and MBCP and their pathway coefficients were explored by constructing structural equation models (**Figure 7**). In the ungrazed grassland, the pathway analysis explained 16% of the variability in the TPCP-MBCP trade-off. The results showed that the model fit well (Lisher’s C = 18.47, *P = 0.06*) RB and APC were positively correlated with the TPCP-MBCP trade-off, and RB and RC indirectly influenced the TPCP-MBCP trade-off through APC. In lightly grazed alpine meadow grassland, the through-trail analysis explained 63% of the variability in the TPCP-MBCP trade-off. The results showed that the model fit well (Lisher’s C = 6.97, *P = 0.137*), RCP was significantly negatively correlated with the TPCP-MBCP trade-off (*P<0.05*), and APCP was positively correlated with the TPCP-MBCP trade-off; RB and RC indirectly affected the TPCP-MBCP trade-off through RCP. In moderately grazed alpine meadow grassland, the through-trail analysis explained 89% of the variability in the TPCP-MBCP trade-off. The results showed that the model fit well (Lisher’s C = 7.644, *P = 0.265*). MBC was significantly positively correlated with the TPCP-MBCP tradeoff (*P<0.05*), and RCP was negatively correlated with the TPCP-MBCP tradeoff; MBC and RC indirectly influenced the TPCP-MBCP tradeoff through APC, and RC indirectly influenced the TPCP-MBCP tradeoff through RCP.

## 4 Discussion

### 4.1 Effect of different levels of litter input on TPCP and MBCP

The Tibetan Plateau is characterized by high altitude and low temperature, and the mean annual temperature is below 0°C in most areas. Under low temperature, soil microorganisms are few in number and low in vitality, resulting in low accumulation of effective nutrients in the soil, and the nutrients required for plant growth and development are supplied by the soil, so that different levels of litter inputs will change the growth and carbon allocation pattern of the plants as well as the storage of the carbon pools. The results of this study showed that ungrazed, lightly grazed and moderately grazed alpine meadow plant biomass, plant carbon content and total plant community carbon pools were positively affected by the amount of apomictic input (*P<0.05*) (**Figures 1**-**3**). Aboveground biomass of alpine meadow plants determined aboveground plant carbon pools, and similarly, root biomass was proportional to the size of root carbon pools. It may be because litter inputs promote root growth because roots need more energy and nutrients to support their uptake and transportation functions, and an increase in both root biomass and content increases RCP as part of the plant (**Figure 7**). Therefore, an increase in both plant biomass and carbon content increase the plant carbon pool.

Microbial biomass carbon (MBC) plays an important role in the soil carbon cycle and is one of the most active and sensitive carbon components in soil. It not only reflects the activity of soil microorganisms, but also affects soil fertility and ecosystem function. The results of this study showed that soil MBC and MBCP in degraded alpine meadows with ungrazed, lightly grazed and moderately grazed grassland were positively affected by litter inputs, and all of them peaked under T3 treatment, which was consistent with the results of the existing studies (Jin et al., 2010; Liu et al., 2019; Pan et al., 2018; Pioli et al., 2020). Maybe high soil microbial biomass is positively correlated with increased litter inputs in nutrient-limited areas (**Figure 5**), most soil microbes, including bacteria and fungi, rely on the decomposition of litter materials for energy and nutrients (Sniegocki et al., 2022). Litter matter serves as an important carbon source for microorganisms in grassland ecosystems. When litter material is inputted into grassland, the organic matter in it is decomposed by microorganisms, thus releasing carbon for microbial utilization, which in turn increases the MBC in the soil. Nevertheless, the content of MBC and MBCP in soil varies depending on the type of grassland, climatic conditions, soil properties and the process of soil microbial anabolism. The present study did not explore the response of MBC and MBCP to meteorological factors and soil factors and the regulatory mechanisms of their interactions on MBC and MBCP, which is a limitation of this study. Follow-up studies should combine meteorological and soil factors to analyze the regulatory mechanisms of MBC and MBCP, which will be conducive to the study of microbial biomass carbon pools in alpine meadows.

### 4.2 Alpine meadows TPCP-MBCP trade-off patterns and regulatory mechanisms

The alpine meadow TPCP-MBCP trade-off model is an adaptation strategy of alpine meadow grassland ecosystems to environmental changes during plant growth. In this study, we found that the ungrazed alpine meadow TPCP-MBCP trade-off (T1 = 0.0414, T2 = 0.0269, and T3 = 0.0012) under litter inputs tended to favor MBCP (**Figure 6**), suggesting that litter inputs contribute to the accumulation of MBCP in ungrazed alpine meadows. The possible reason is that when fresh carbon is input to the soil, it stimulates the activity of soil microorganisms and the initiating effect accelerates the decomposition and mineralization of soil organic matter. The present study area belongs to grassland degraded alpine meadows with low soil organic matter content, the initiation effect may be more significant and the microbial activity may be more intense, which leads to the increase of soil microbial biomass carbon, and consequently increases MBCP.

The results of this study showed that the TPCP-MBCP trade-offs of lightly and moderately grazed alpine meadows tended to favor TPCP as a whole (T2 = 0.0494 and T3 = 0.0383, respectively), and that litter inputs contributed to the accumulation of TPCP in lightly and moderately grazed alpine meadows. This may be due to the high altitude and low air temperature in the study area, and the mean annual temperature of the study area was below 0 °C, and the soil microorganisms had fewer species and numbers and lower vigor in the low-temperature condition, which resulted in less accumulation of effective nutrients in the soil. Litter inputs can supplement soil nutrients and alleviate soil nutrient deficiencies during the reproductive growth stage of plants. Grassland degradation in the Tibetan Plateau is serious, the primary productivity of vegetation is reduced, and the large amount of soil and water loss leads to soil infertility. Dwarf tarragon, as the main establishment species of alpine meadow plant communities, has a short plant coefficient, and the root competition is more intense than the aboveground competition, and the plant transfers the productivity to the roots to use it for competing for resources (Kiær et al., 2013). At the same time, plants use the synthesized photosynthesis products for more than their own growth, and transport excess photosynthesis products to the root system for storage (**Figure 7**). TPCP as the sum of both APCP and RCP. Therefore, the alpine meadow TPCP-MBCP trade-off as a whole favors plant communities during the peak plant growth season.

Variations in the TPCP-MBCP trade-off in alpine meadows differed between different levels of litter inputs. In this study, it was found that the TPCP-MBCP trade-off favored MBCP under ungrazed, lightly grazed, and moderately grazed under T1 treatment (**Figure 6**). This is consistent with the results of an existing study (Wei et al., 2022). It is possible that litter inputs initiated the excitation effect of soil carbon, which affected the acquisition and competition between plants and microorganisms for months, and the TPCP-MBCP trade-off was subjected to dual regulatory pathways (i.e., *in vivo* modification and *in vitro* turnover) by soil microorganisms (Liang and Zhu, 2021). Differences in soil microbial community structure and functional genes modulate soil organic matter decomposition and accumulation driving soil microbial assimilation. Meanwhile, alpine meadows form a certain equilibrium between alpine meadow plants and microorganisms thought the stability of grassland ecosystems due to nutrient deficiencies, so that a small amount of litter inputs in the short term contributes to the accumulation of MBC, which leads to the TPCP-MBCP trade-off close to MBCP.

## 5 Conclusion

Plant biomass, plant carbon content and MBC and MBCP in ungrazed, lightly grazed and moderately grazed alpine meadows were positively affected by the amount of litter input. Under different levels of litter treatments, the trade-off relationship of TPCP-MBCP in ungrazed alpine meadow was in the order of T1 (0.0414) > T2 (0.0269) > T0 (0.0086) > T3 (0.0012), and the trade-off relationship of TPCP-MBCP in lightly grazed alpine meadow was in the order of T2 (0.0494) > T3 (0.0140) > T0 (0.0097) > T1 (0.002), and the trade-off relationship of TPCP-MBCP in moderately grazed alpine meadows was in the order T3 (0.0383) > T1 (0.0307) > T2 (0.0196) > T0 (0.0005). The propensity analysis showed that the TPCP-MBCP trade-off under ungrazed, lightly grazed and moderately grazed under T1 treatment tended to favor MBCP. The present study is helpful to understand the effects of different grazing degraded alpine meadows on the TPCP-MBCP trade-off under litter inputs, to optimize the grazing management strategy and to ensure the sustainable development of the Qinghai-Tibetan Plateau.

## Data Availability

The original contributions presented in the study are included in the article/supplementary material. Further inquiries can be directed to the corresponding author.
